# Na^+^/Ca^2+^ exchanger 1 on nuclear envelope controls PTEN/Akt pathway via nucleoplasmic Ca^2+^ regulation during neuronal differentiation

**DOI:** 10.1038/s41420-017-0018-1

**Published:** 2018-04-30

**Authors:** Agnese Secondo, Alba Esposito, Tiziana Petrozziello, Francesca Boscia, Pasquale Molinaro, Valentina Tedeschi, Anna Pannaccione, Roselia Ciccone, Natascia Guida, Gianfranco Di Renzo, Lucio Annunziato

**Affiliations:** 1 IRCCS SDN, School of Medicine, “Federico II” University of Naples, Naples, Italy; 2 IRCCS SDN, Naples, Italy

## Abstract

Nuclear envelope (NE) is a Ca^2+^-storing organelle controlling neuronal differentiation through nuclear Ca^2+^ concentrations ([Ca^2+^]_n_). However, how [Ca^2+^]_n_ regulates this important function remains unknown. Here, we investigated the role of the nuclear form of the Na^+^/Ca^2+^ exchanger 1(nuNCX1) during the different stages of neuronal differentiation and the involvement of PTEN/PI3’K/Akt pathway. In neuronal cells, nuNCX1 was detected on the inner membrane of the NE where protein expression and activity of the exchanger increased during NGF-induced differentiation. nuNCX1 activation by Na^+^-free perfusion induced a time-dependent activation of nuclear-resident PI3K/Akt pathway in isolated nuclei. To discriminate the contribution of nuNCX1 from those of plasma membrane NCX, we generated a chimeric protein composed of the fluorophore EYFP, the exchanger inhibitory peptide, and the nuclear localization signal, named XIP-NLS. Fura-2 measurements on single nuclei and patch-clamp experiments in whole-cell configuration showed that XIP-NLS selectively inhibited nuNCX1. Once it reached the nuclear compartment, XIP-NLS increased the nucleoplasmic Ca^2+^ peak elicited by ATP and reduced Akt phosphorylation, GAP-43 and MAP-2 expression through nuclear-resident PTEN induction. Furthermore, in accordance with the prevention of the neuronal phenotype, XIP-NLS significantly reduced TTX-sensitive Na^+^ currents and membrane potential during neuronal differentiation. The selective inhibition of nuNCX1 by XIP-NLS increased the percentage of β III tubulin-positive immature neurons in mature cultures of MAP-2-positive cortical neurons, thus unraveling a new function for nuNCX1 in regulating neuronal differentiation through [Ca^2+^]_n_-dependent PTEN/PI3K/Akt pathway.

## Introduction

Nuclear Ca^2+^ concentrations ([Ca^2+^]_n_) regulate many cellular functions by modulating specific proteins^1^. For instance, the nuclear-targeted Ca^2+^ buffer protein parvalbumin, but not the cytosolic isoform, reduces hepatocyte proliferation^2^. Moreover, the activation of the transcription factors Elk1^3^, NFAT, DREAM^4^, and CREB depends on nuclear Ca^2+^ levels. On the other hand, intranuclear Ca^2+^ dysregulation may affect cell survival, as it occurs under  hypoxic conditions where it triggers the expression of pro-apoptotic genes^5,6^. [Ca^2+^]_n_ is influenced by cytosolic Ca^2+^ levels and is regulated by the activation of specific receptors, pumps, and exchangers localized on the nuclear envelope (NE)^7,8^. For instance, the stimulation of nuclear inositol (1,4,5)-triphosphate receptors (InsP_3_R) leads to nucleoplasmic Ca^2+^ increase in isolated nuclei from Xenopus oocytes^9^, Aplysia neurons^10^, and pancreatic acinar cells^11^. Furthermore, the activation of G-protein coupled receptors localized on the NE modulates—via [Ca^2+^]_n_—gene transcription, import and export pathways of proteins, and cell cycle^12^. In addition, outer and inner membranes of NE are provided with K^+^, Cl^−^, and R-type Ca^2+^ channels^13–15^, Na^+^/H^+^ exchanger 1^16^, Ca^2+^-ATPase pump^17^, and NCX1^18,19^. Despite the existent knowledge on the role of plasma membrane NCX in the regulation of intracellular Ca^2+^ concentrations and Ca^2+^-dependent pathways^20–22^, nuNCX1 function remains unexplored. Interestingly, global silencing of NCX1 prevents neurite outgrowth through the inhibition of Akt pathway thus reducing NeuN-positive neurons^23^. On the other hand, it has been reported that miR-29a targeting the phosphatase and tensin homologs on chromosome ten (PTEN), increase phosphorylation of Akt, thus promoting neurite outgrowth^24^. Indeed, deregulation of PTEN affects neurogenesis, neurite outgrowth, synaptogenesis, and synaptic plasticity^25,26^. Therefore, the purpose of the present study has been to investigate the contribution of nuNCX1 in the modulation of nuclear-resident PTEN/PI3K/Akt pathway via [Ca^2+^]_n_ during neuronal differentiation.

## Results

### Characterization of nuNCX1 expression in neuronal cells

**Fig. 1:**
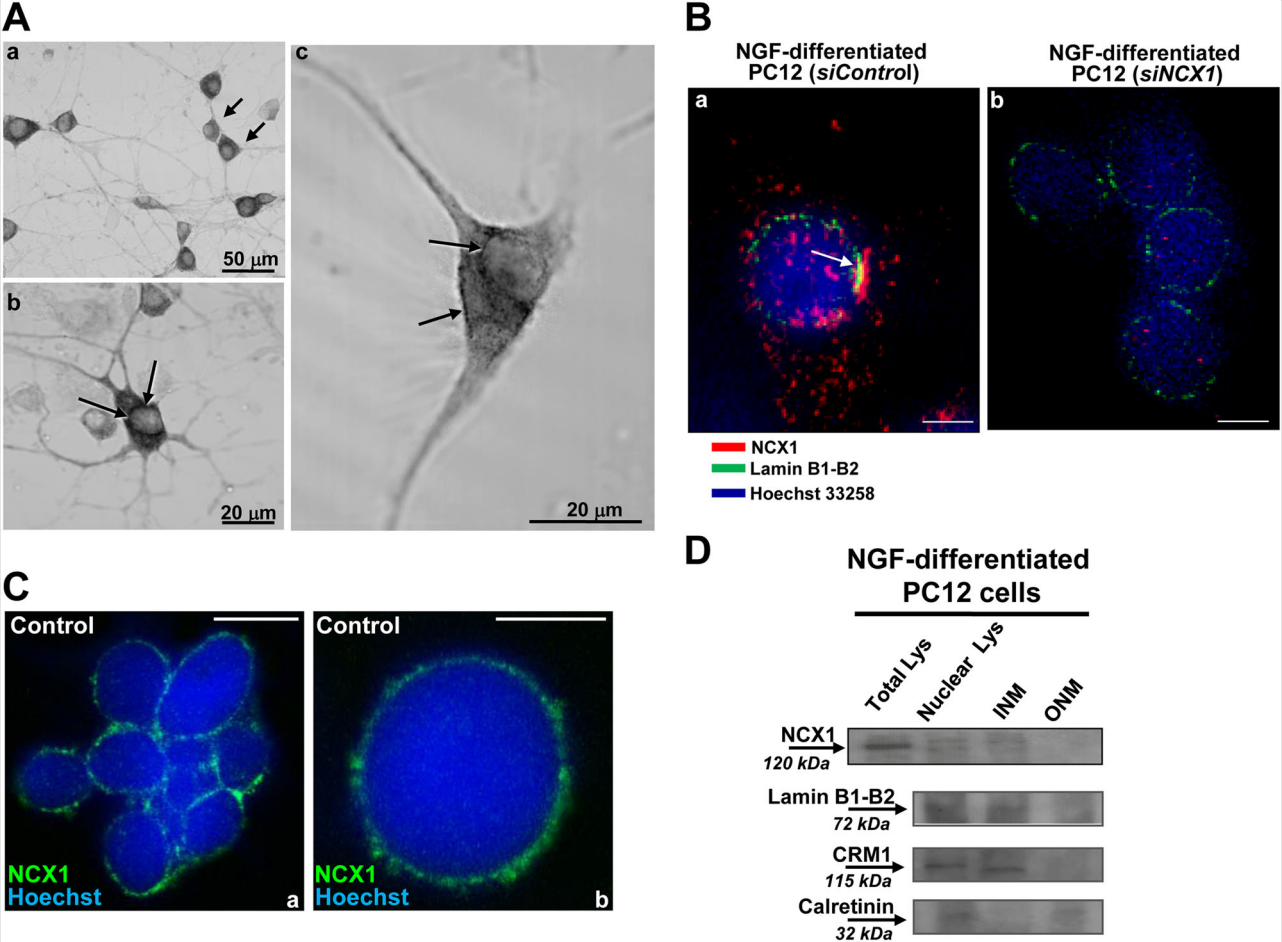
Localization of neuronal Na^+^/Ca^2+^ exchanger 1 at the inner membrane of the NE. **a** Immunolocalization of NCX1 in whole cell obtained by 3,3′-diaminobenzidine in differentiated PC12 cells treated with NGF for 7 days. Scale bars: 50 µm (a), 20 µm (b, c). **b** Co-localization of NCX1 (red) with Lamin B1/B2 (green), the inner membrane of the NE marker, in differentiated PC12 cells detected by confocal microscopy under control conditions treated with siControl (a) and in NCX1-silenced PC12 cells (b). Scale bars: 5 µm. **c** Confocal images representing immunofluorescent signal of NCX1 (green) and Hoechst (blue) in isolated nuclei from differentiated PC12 cells under control conditions (a, b). **d** Western blot of NCX1, Lamin B1/B2, CRM1 and calretinin in total and nuclear lysates, inner and outer NE membranes of NGF-differentiated PC12 cells. All the experiments were repeated at least three times on different preparations

Immunolocalization of the NCX1 isoform in NGF-differentiated PC12 cells revealed diffused DAB immune-staining of this isoform. Together with its plasmamembrane expression, NCX1 was also present at the nuclear level (Fig. 1Aa, b, c). Furthermore, in specific NE areas, NCX1 co-localized with Lamin B1/B2 (Fig. 1Ba), a component of nuclear lamina connected with the nucleoplasmic face of the inner membrane (INM). However, when NGF-differentiated PC12 cells were transfected with siRNA against NCX1 (siNCX1), Lamin B1/B2-NCX1 co-localization disappeared (Fig. 1Ba, c). Moreover, in whole neuronal cells, NCX1 immunosignal was also found outside the nucleus (Fig. 1Ba) and disappeared in cells treated with siNCX1 (Fig. 1Bb). Confocal analysis showed a significant immunosignal of NCX1 on the NE of isolated nuclei derived from differentiated PC12 cells (Fig. 1Ca, b). Nissl staining showed the lack of ER contamination and the integrity of isolated nuclei while ethidium bromide staining confirmed the high purity of these preparations and the lack of dispersed DNA (data not shown). In addition, any possible contamination by specific markers of the plasmamembrane, mitochondria, Golgi complex, and ER such as the plasma membrane calcium ATPase, Na^+^ channels, dynamin-related protein 1, GM130, and stromal interaction molecule 1 were detected in the preparation of isolated nuclei, except for the two nuclear markers H3 and Akt (data not shown).

To further confirm the specific localization of NCX1 at INM level, we performed a separation between outer and inner membranes of NE with cold Na-citrate in isolated nuclei obtained from NGF-differentiated PC12 cells. Once the outer membrane was removed, NCX1 was detected by Western blot only on the inner membrane attached to the nuclei and in total lysate of both whole cells and total nuclei (Fig. 1D). Efficient isolation of INM was confirmed by the expression of Lamin B1/B2 and CRM1, a member of nuclear transport factor family (Fig. 1D). Furthermore, only the preparation of outer NE membrane expressed calreticulin, an ER/SR protein that is contiguous with the outer NE (Fig. 1D).

### Expression and function of NCX isoforms in NE

**Fig. 2:**
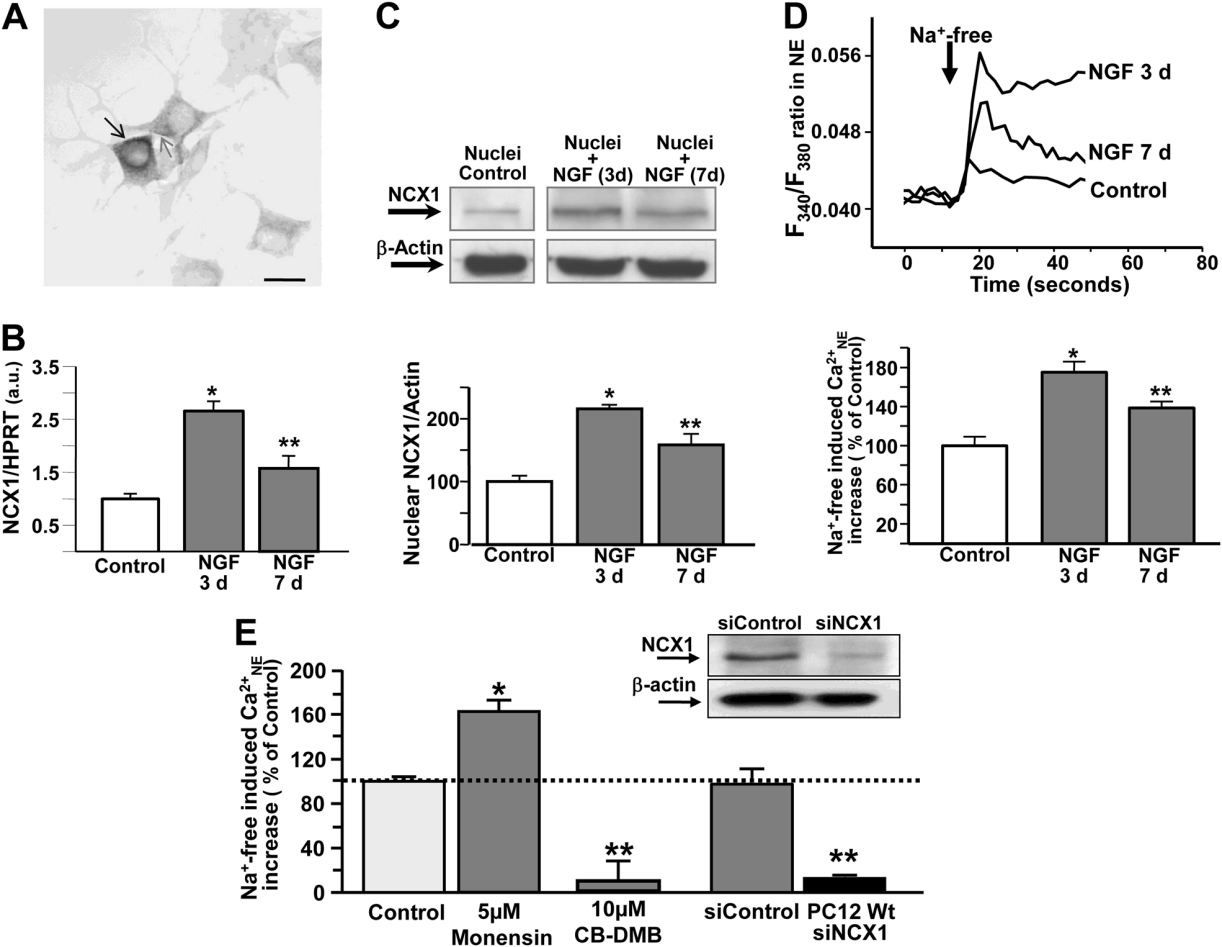
nuNCX1 expression and activity in nuclei from PC12 cells during differentiation with NGF. **a** Immunolocalization of NCX1 in whole cell obtained by 3,3′-diaminobenzidine in PC12 cells treated with NGF for 3 days. Scale bars: 50 µm (a), 20 µm (b). **b** Representative qRT-PCR of *ncx1* transcript expression in control cells and in PC12 cells after 3 and 7 days exposure to NGF. **c** Representative Western blot and quantification of nuNCX1 protein expression in isolated nuclei from PC12 cells in control conditions and after 3 and 7 days exposure to NGF. For **b** and **c**: all the experiments were repeated at least three times on different nuclear preparations; *p < 0.05 vs. undifferentiated control, **p < 0.05 vs. undifferentiated control and 3 days NGF. **d** Top: Representative traces of the effect on [Ca^2+^]_n_ of Na^+^-free perfusion in Fura-2-loaded nuclei isolated from undifferentiated PC12 cells (control), and PC12 cells exposed to 3 days or 7 days to NGF. Bottom: quantification of the effect of **d**. *p < 0.001 vs. undifferentiated control, **p < 0.05 vs. undifferentiated control and 3 days NGF. **e** Quantification of the effect on [Ca^2+^]_n_ of Na^+^-free in isolated nuclei obtained from NGF-differentiated PC12 cells pre-treated with: monensin (10 min), CB-DMB (20 min), and siControl or siNCX1 (at DIV 5). *p < 0.001 vs. undifferentiated control, **p < 0.001 vs. all groups. Inset: Representative Western blot depicts the effect of siControl and siNCX1 on nuNCX1 protein expression in isolated nuclei from NGF-differentiated PC12 cells

In an attempt to study the involvement of nuNCX1 in the different stages of neuronal differentiation, immunolocalization of this isoform was performed also in PC12 cells exposed to NGF for 3 days before complete differentiation. At 3 days, DAB immunostaining of NCX1 was almost totally detected in nuclear compartment (Fig. 2A, black arrow) and slightly localized in some part of plasma membrane (Fig. 2A, gray arrow). qRT-PCR experiments showed that, during differentiation with NGF, NCX1 transcripts increased in PC12 cells with a significant peak at 3 days of exposure (Fig. 2B). On the other hand, the transcripts of the third isoform of the exchanger, NCX3, significantly reduced at 3 and 7 days of exposure (in A.U. ± SEM: control = 1.02 ± 0.03; 3 days NGF = 0.52 ± 0.05 *p < 0.05 vs. control; 7 days NGF = 0.55 ± 0.08, *p < 0.05 vs. control). Moreover, Western blot analysis revealed that nuNCX1 expression in PC12 cells peaked after 3 days exposure to NGF in nuclear preparations (Fig. 2C), thus suggesting a specific role played by nuclear NCX1 in triggering differentiation at that time. By contrast, NCX3 protein expression dropped dramatically in the same preparation (data not shown).

Then, NCX1 activity was studied by exposing to a Na^+^-free solution single nuclei loaded with Fura-2AM, a fluorescent dye exclusively concentrated in NE lumen^27^. Na^+^-free perfusion forced the exchanger to operate in the forward mode, moving Ca^2+^ from the nucleoplasm to the NE lumen. This elicited an increase of Ca^2+^ level in the NE lumen. In particular, this [Ca^2+^] increase induced by Na^+^-free exposure was much higher in nuclei obtained from PC12 cells exposed to NGF for 3 days than those exposed for 7 days or undifferentiated controls (Fig. 2D). In the same nuclear preparation, a pharmacological characterization of NCX1 activity in the forward mode was performed by the Na^+^ ionophore monensin, or blocking NCX by the amiloride analog 3-amino-6-chloro-5-[(4-chloro-benzyl)amino]-n-[[(2,4-dimethylbenzyl)-amino]iminomethyl]-pyrazinecarboxamide (CB-DMB)^28^. Whereas monensin was able to enhance the Na^+^-dependent activity of the exchanger, possibly by increasing intra-nuclear Na^+^ levels, CB-DMB prevented the increase in NE Ca^2+^ level associated with NCX activation (Fig. 2E). Furthermore, NE Ca^2+^ increase was completely prevented in isolated nuclei from PC12 cells previously treated with siNCX1(Fig. 2E).

### nuNCX1 silencing prevents nuclear-resident Akt phosphorylation induced by Na^+^-free perfusion

**Fig. 3:**
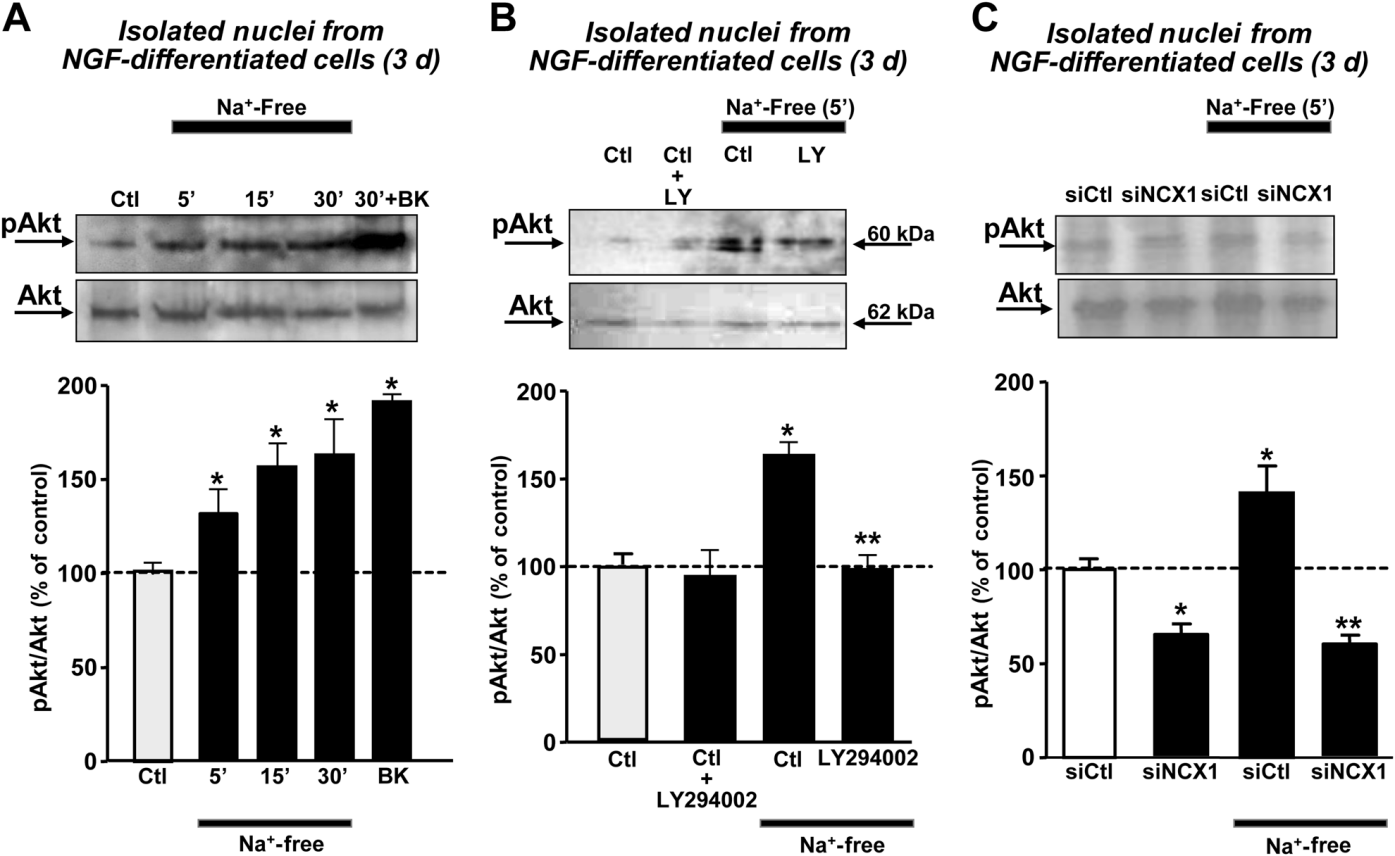
Nuclear-resident Akt activation by Na^+^-free exposure in nuclear preparations from neuronal cells. **a** Representative Western blot and quantification of phosphorylated Akt in isolated nuclei from NGF-differentiated PC12 cells (3 days) after perfusion with Na^+^-free or bradykinin (BK). **b** Representative Western blot and quantification of phosphorylated Akt in isolated nuclei from NGF-differentiated PC12 cells previously treated with the PI3’K inhibitor LY294002 **c** Representative Western blot and quantification of the effect of Na^+^-free (5 min) in isolated nuclei obtained from NGF-differentiated PC12 cells (3 days) previously treated with siControl or siNCX1. For **a**, **b**, **c**: all the experiments were repeated at least three times on different nuclear preparations. *p < 0.05 vs. untreated control or siControl, both not exposed to Na^+^-free. **p < 0.05 vs. control or siControl + 5′ Na^+^-free

To verify whether nuNCX1 activation could modulate the phosphorylation of nuclear-resident Akt, a Ca^2+^-dependent kinase mainly involved in neurite outgrowth, we exposed isolated nuclei to a Na^+^-free solution under controlled conditions of temperature. Isolated nuclei were obtained from PC12 cells treated with NGF for 3 days, a time of the highest expression of nuNCX1 (see Fig. 2). Na^+^-free induced phosphorylation of nuclear-resident Akt in isolated nuclei from PC12 cells (Fig. 3a). Moreover, Na^+^-free-induced Akt phosphorylation was evident already after 5 min of exposure (Fig. 3a). The same activation of Akt was induced by exposing intact nuclei to bradykinin (BK) (Fig. 3a), whose Gq-coupled receptors are located on the NE inner membrane^29^. This effect was prevented by pre-treating nuclei for 10 min with the PI3′-K inhibitor LY294002 (25 µM) before Na^+^-free exposure (Fig. 3b). Interestingly, in nuclear preparations obtained from neuronal cells previously exposed to siNCX1, Na^+^-free perfusion (5 min) failed to induce Akt phosphorylation compared to siControl cells (Fig. 3c).

### Selective inhibition of nuNCX1 by the chimeric protein XIP-NLS increases nucleoplasmic Ca^2+^ level thus limiting neuronal differentiation

**Fig. 4:**
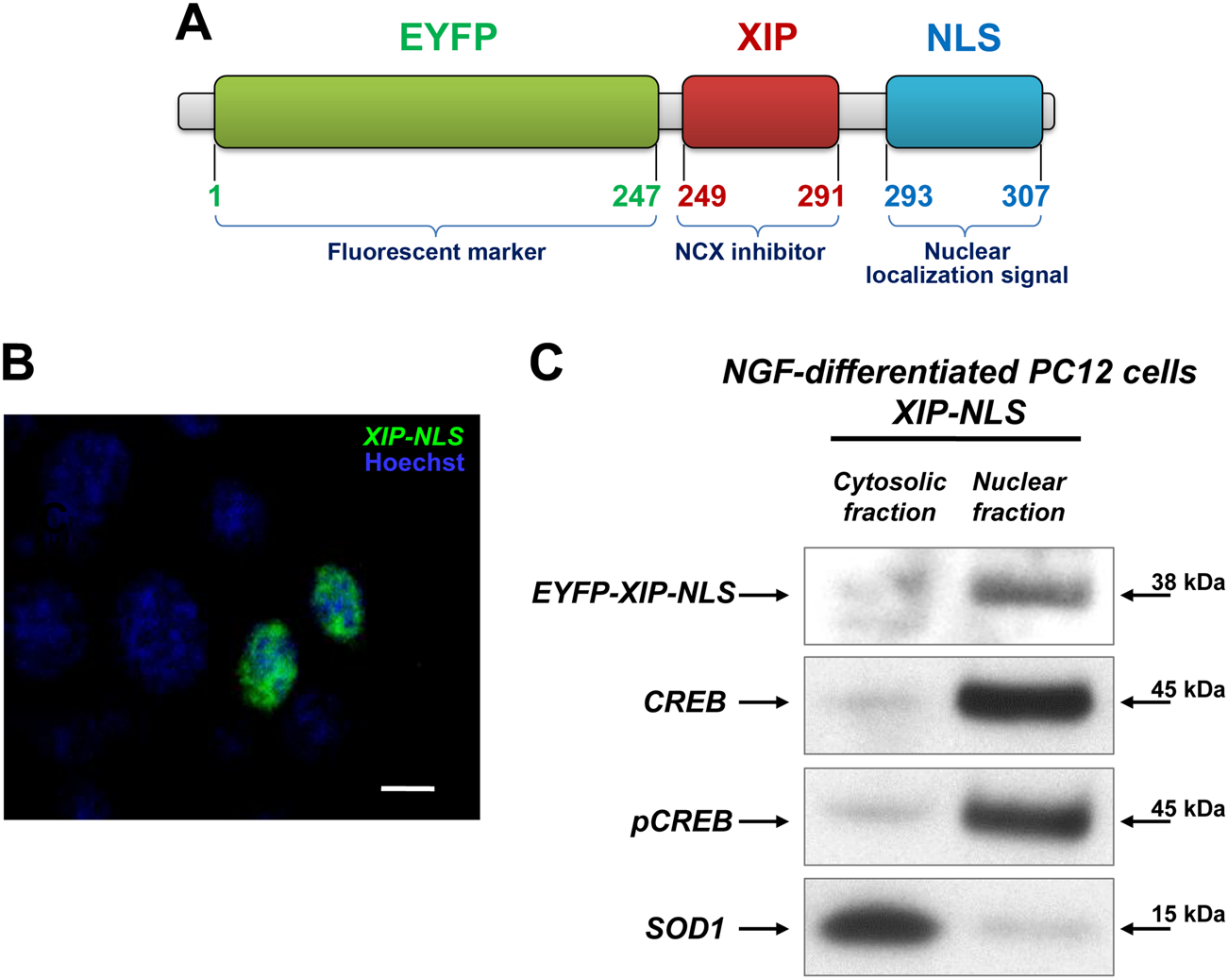
Subcellular localization of the chimeric protein XIP-NLS. **a** Scheme of XIP-NLS structure. **b** Immunolocalization of XIP-NLS (green, 400 ng/ml) detected 48 h after transfection. Hoechst dye (blue) was used to mark nuclei. Under these conditions, EYFP-positive nuclei were at least 25 ± 2% of total Hoechst-positive nuclei. **c** Western blot of EYFP, CREB, pCREB, and SOD1 in cytosolic and nuclear fractions obtained from NGF-differentiated PC12 cells transfected with XIP-NLS (400 ng/ml). Fractions were prepared 48 h after transfection

Forty-eight hours after the transfection of XIP-NLS (Fig. 4a), confocal microscopy and immunoblot analysis revealed that XIP-NLS was selectively localized in the nuclei of NGF-differentiated PC12 cells (Figs. 4b, c). Indeed, EYFP signal co-localized with nuclear DNA staining Hoechst 33258 in nuclei efficiently transfected with XIP-NLS (Fig. 4b). Furthermore, the specificity of the nuclear localization of XIP-NLS was revealed by the co-detection, after 48 h, with the nuclear markers CREB and pCREB in the same fraction. Further evidence for the nuclear localization of XIP-NLS and for the purity of our cellular fractions was the absence of the cytosolic protein SOD1 in the nuclear fraction (Fig. 4c).

**Fig. 5:**
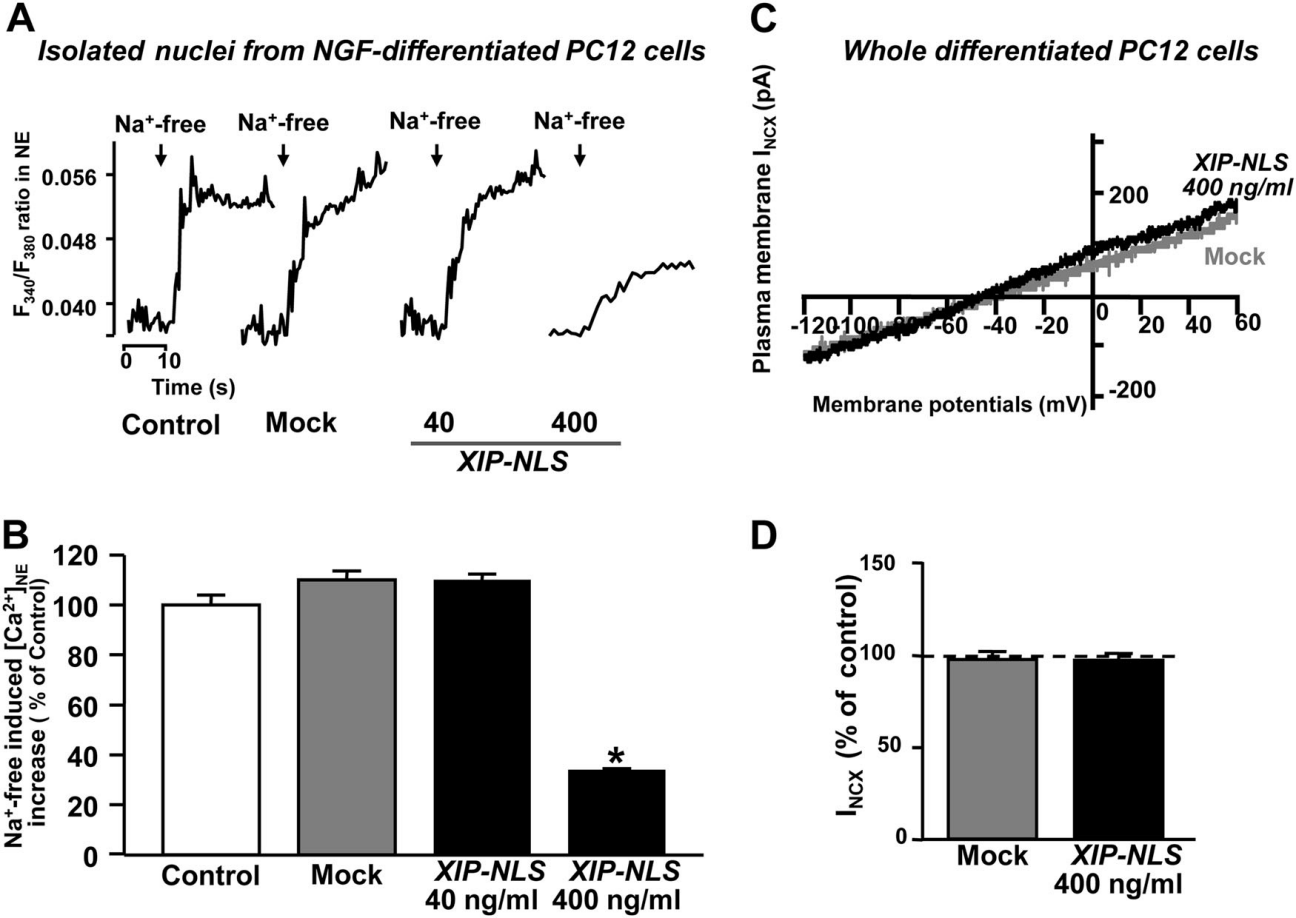
Functional characterization of the chimeric protein XIP-NLS. **a** Representative traces of Fura-2-detected [Ca^2+^]_n_ after Na^+^-free perfusion in isolated nuclei prepared from NGF-differentiated PC12 cells previously transfected with mock or XIP-NLS. **b** Quantification of **a**. *p < 0.001 vs. all. Control (*n* = 15 cells); mock (*n* = 20 cells); XIP-NLS 40 (*n* = 15 cells); XIP-NLS 400 (*n* = 15 cells). **c** Superimposed traces of I_NCX_ currents recorded by patch-clamp in NGF-differentiated PC12 cells previously transfected with mock or XIP-NLS. **d** Quantification of **c**. mock (*n* = 20 cells); XIP-NLS (*n* = 20 cells)

Furthermore, XIP-NLS inhibited Na^+^-dependent nuNCX1 activity elicited by Na^+^-free perfusion in isolated nuclei obtained from NGF-differentiated PC12 cells, as detected by Fura-2 microfluorimetry (Figs. 5a, b). By contrast, as expected by the nuclear selectivity of the chimeric protein, XIP-NLS did not affect NCX currents measured by patch-clamp at plasma membrane level in NGF-differentiated PC12 cells (Figs. 5c, d).

**Fig. 6:**
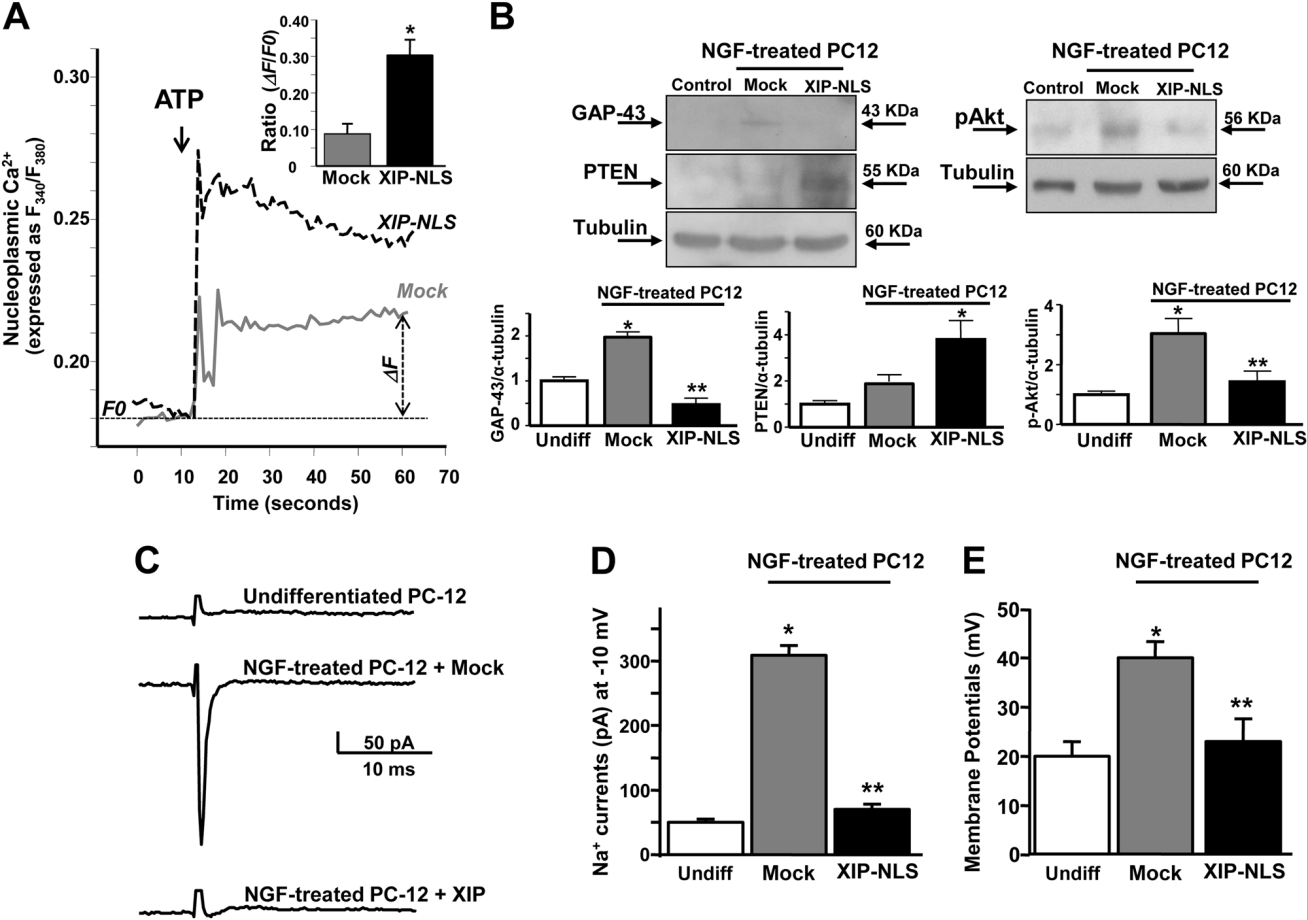
Effect of the chimeric protein XIP-NLS on [Ca^2+^]_n_ and neuronal differentiation in NGF-treated PC12 cells. **a** Representative traces of Fura-dextran-detected nucleoplasmic Ca^2+^ after ATP (100 µM) perfusion in NGF-treated PC12 cells (3 days) previously transfected with mock or XIP-NLS. Bar graph depicts quantification of ATP-induced nucleoplasmic Ca^2+^ increase. *p < 0.05 vs. mock. mock (*n* = 20 cells); XIP-NLS (*n* = 15 cells). **b** Representative Western blots and quantifications of GAP-43, PTEN protein expression, and Akt phosphorylation in NGF-treated PC12 cells (3 days) previously transfected with mock or XIP-NLS. The experiments were repeated at least three times on different preparations; *p < 0.05 vs. undifferentiated cells; **p < 0.05 vs. mock. **c** and **d** Representative traces and quantification  of voltage-gated sodium currents (I_NaV_) recorded from PC12 cells under control conditions (undifferentiated, *n* = 6), after exposure to NGF for 3 days and mock transfection (*n* = 10) and after exposure to NGF for 3 days and NLS-XIP transfection (*n* = 20). The normalization for membrane capacitances produced similar results. **e** Quantification of membrane potential in all the conditions aforementioned. *p < 0.001 vs. undifferentiated cells; **p < 0.001 vs. NGF-treated PC12 cells + Mock

In NGF-treated PC12 cells transfected with XIP-NLS, this peptide significantly increased Fura-dextran-detected nucleoplasmic Ca^2+^ peak in response to the Gq-protein coupled receptor agonist ATP (100 µM) compared to mock control (Fig. 6a). Once transfected, XIP-NLS significantly reduced GAP-43 expression and Akt phosphorylation in PC12 cells exposed to NGF for 3 days compared to mock control (Fig. 6b). Interestingly, under the same conditions, XIP-NLS significantly increase the expression of the negative regulator of PI3K/Akt pathway, phosphatase and tensin homolog (PTEN) (Fig. 6b), mainly involved in axonal growth modulation^24^. Then, in accordance with the prevention of the neuronal phenotype, XIP-NLS significantly reduced TTX-sensitive Na^+^ currents in PC12 cells exposed to NGF for 3 days (Figs. 6, d). Furthermore, XIP-NLS significantly decreased membrane potential in PC12 cells exposed to NGF for 3 days compared to the respective controls (Fig. 6e).

**Fig. 7:**
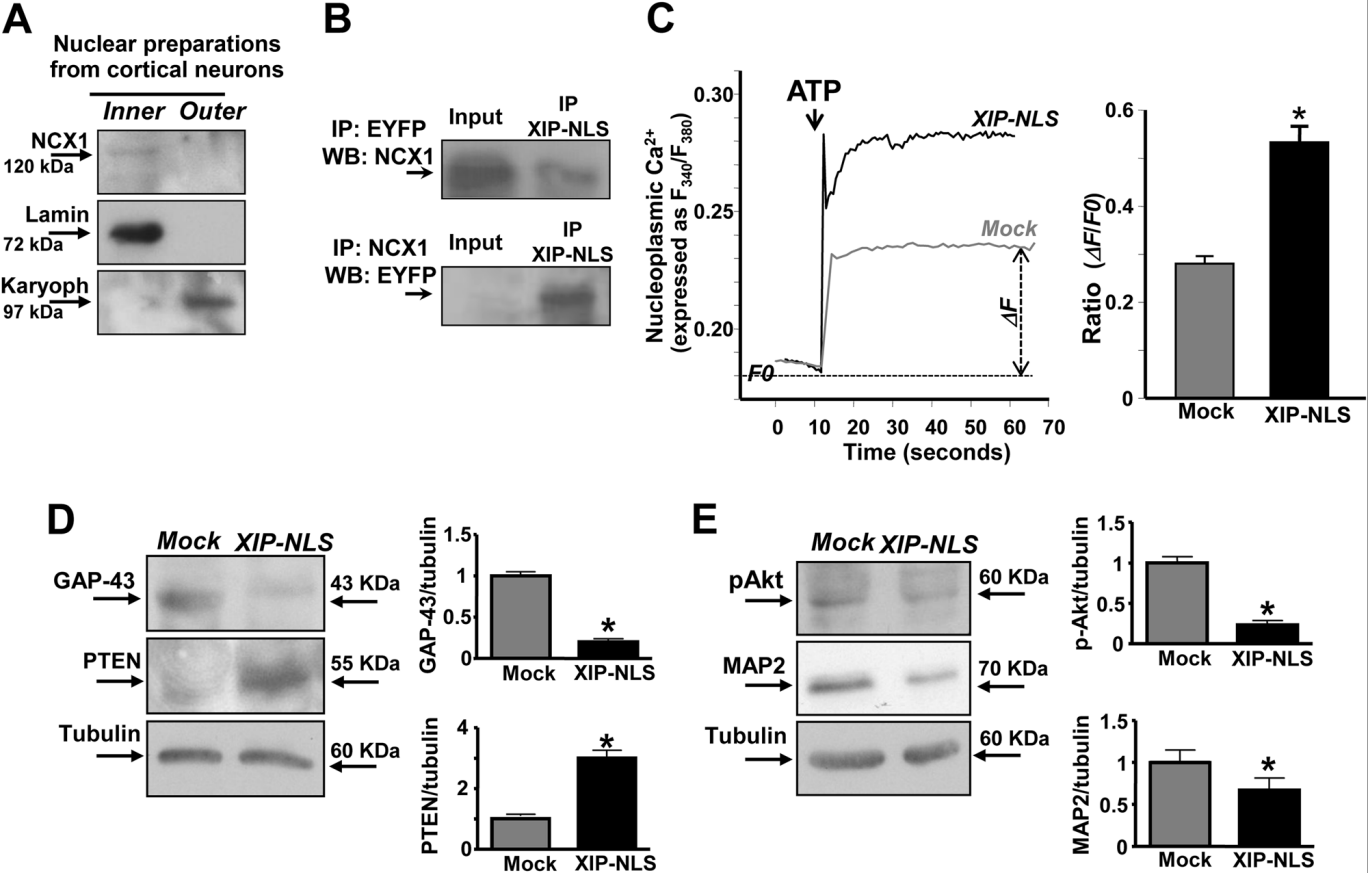
Effect of the chimeric protein XIP-NLS on [Ca^2+^]_n_ and neuronal differentiation in primary cortical neurons. **a** Western blot of NCX1, Lamin B1/B2, and karyopherin in inner and outer NE membranes of cortical neurons at 7 DIV. All the experiments were repeated at least three times on different preparations. **b** Nuclear lysates from neurons trasfected with XIP-NLS (48 h) and immunoprecipitated with anti-EYFP (top) or anti-NCX1 (bottom). The presence of NCX1 (top) or EYFP (bottom) was analyzed by immunoblotting performed with specific antibodies. The input consists on nuclear lysates from untreated neurons at 7 DIV. **c** Representative traces of Fura-dextran-detected nucleoplasmic Ca^2+^ after ATP (100 µM) perfusion in cortical neurons previously transfected with mock or XIP-NLS. Bar graph depicts quantification of ATP-induced nucleoplasmic Ca^2+^ increase. *p < 0.05 vs. mock. mock (*n* = 15 cells); XIP-NLS (*n* = 10 cells). **d**, **e** Representative Western blots and quantifications of GAP-43, MAP-2, PTEN protein expression, and Akt phosphorylation in cortical neurons (7DIV) previously transfected with mock or XIP-NLS. All the experiments were repeated at least three times on different preparations; *p < 0.05 vs. mock

**Fig. 8:**
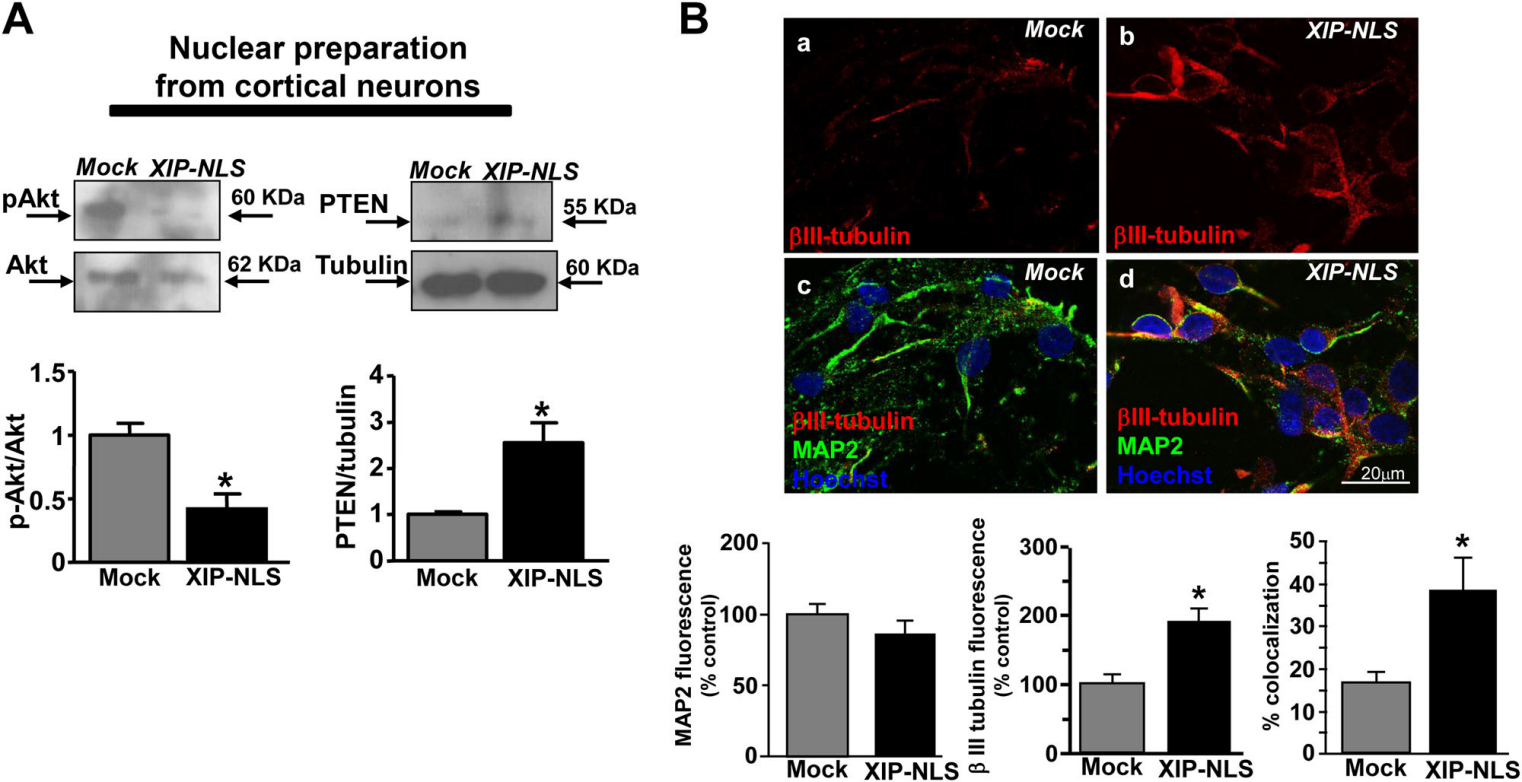
Effect of nuclear NCX1 inhibition on nuclear-resident PTEN/Akt pathway and on βIII-tubulin and MAP-2 immunostaining in mature cortical neurons. **a** Representative Western blots and quantifications of PTEN expression and Akt phosphorylation in nuclear preparations from mature cortical neurons (7DIV) transfected at 5 DIV with mock or XIP-NLS. All the experiments were repeated at least three times on different preparations. *p < 0.001 vs. mock. **b** Top: βIII-tubulin and MAP-2 staining in cortical neurons at 7 DIV under control conditions and in neurons transfected with XIP-NLS (bar = 20 μm; nuclei Hoechst blue). Bottom: Quantification of MAP-2 and βIII-tubulin fluorescence and % of their colocalization in cortical neurons at 7 DIV

A separation between outer and inner NE membranes of isolated nuclei obtained from primary cortical neurons showed a specific localization of NCX1 on the inner membrane attached to the nuclei (Fig. 7a). Furthermore, efficient isolation of INM in cortical neurons was confirmed by the expression of Lamin B1/B2 (Fig. 7a). On the other hand, the preparation of outer NE membrane expressed karyopherin β2 (Fig. 7a). Interestingly, once transfected at higher concentrations in cortical neurons, XIP-NLS carrying EYFP co-immunoprecipitated with nuNCX1 as detected with anti-NCX1 in isolated nuclei (Fig. 7b). As occurred in NGF-treated PC12 cells, XIP-NLS significantly increased Fura-dextran-detected nucleoplasmic Ca^2+^ peak in response to ATP (100 µM) in cortical neurons (Fig. 7c). This suggests that XIP-NLS was able to reduce Ca^2+^ buffering properties of neuronal nuclei. Moreover, also in mature neurons (7 DIV), the chimeric protein reduced terminal differentiation markers, such as GAP-43 and MAP-2 (Figs. 7d, e), significantly increased PTEN expression and decreased Akt phosphorylation not only in total lysates (Fig. 7d, e) but also in isolated nuclei (Fig. 8a) from cortical neurons. In order to strength the evidence demonstrating the role of nuclear NCX1 isoform in the differentiation of primary cortical neurons, we analyzed the effect of the nuclear inhibitor of NCX1, XIP-NLS, on the markers of both immature and mature rat cortical neurons. To this aim we performed confocal double immunofluorescence experiments with the anti-βIII-tubulin, a marker of immature neurons, and microtubule-associated protein-2 (MAP-2) that is expressed throughout all stages of neuronal differentiation. We found that the selective inhibition of nuclear NCX1 by transfecting XIP-NLS increased the percentage of βIII-tubulin-positive immature neurons after 7 DIV compared with control cultures but did not change the percentage of MAP-2-positive neurons (Fig. 8b).

## Discussion

The present study demonstrates that the nuclear Na^+^/Ca^2+^ exchanger isoform 1 (nuNCX1), located at the level of the inner membrane of NE (INM) in neurons, plays a relevant role in neuronal differentiation through the modulation of nuclear Ca^2+^ homeostasis. However, we found NCX1 immunosignal also outside the INM, possibly at the level of other intracellular organelles, whereas no detectable traces of the other two NCX isoforms, NCX2 and NCX3 were detected on INM. Compelling evidence for NCX1 specific localization to the INM is that, in nuclear preparations obtained from cortical neurons in which outer membrane was removed, nuNCX1 was co-expressed with specific markers of the INM, namely laminB1/B2 and CRM1. Furthermore, this specific localization was paralleled by its specific activation. In particular, working in the forward mode of operation, nuNCX1 moved Ca^2+^ from the nucleoplasm to the NE lumen of neuronal cells. Indeed, NCX1 silencing, as well as the pharmacological inhibition of its activity by the amiloride derivative CB-DMB, abrogated Ca^2+^ rise in the lumen of NE due to the forward mode of the nuclear exchanger, thus demonstrating that nuNCX1 has the same pharmacological sensitivity of its plasmamembrane form. Therefore, nuNCX1 contributes to the Na^+^-dependent removal of [Ca^2+^] from nucleoplasm of neuronal cells. In this regard, we speculated that Ca^2+^ extrusion from the total nuclear compartment of a whole cell might entail two consecutive steps: the first step, operated by NCX1 located on the inner membrane of the NE, might mediate Ca^2+^ transport from the nucleoplasm to the lumen of the NE^30^, and the second step, whose transporter mechanism is still unknown, might promote Ca^2+^ movement from the lumen of the nuclear envelope into the ER. Such new function might be supported by the presence of a Na^+^ gradient existing between the lumen of the nuclear envelope and the nucleoplasm. Consistently, previous evidence indicates the presence of a Na^+^ concentration gradient between the lumen (80 mM) and the nucleoplasm (5–20 mM), that is maintained by the nuclear form of the Na^+^/K^+^ ATPase^31^. Indeed, this pump is present in the inner membrane of the nuclear envelope and is oriented with the ATP hydrolysis site in the nucleoplasm^27,31^.

Indeed, the present finding is in line with our previous results demonstrating that NCX1 participates in NGF-induced neuronal differentiation via nuclear Akt^23^. However, the present data seems to suggest that, among the different exchangers, nuNCX1 plays a major role in inducing neuronal differentiation working in the early phase of the process. Consistently, nuNCX1 expression and activity progressively increased during differentiation, thus peaking at 3 days of NGF exposure. At this time, nuNCX1 mainly controls nuclear-resident PTEN/Akt pathway via [Ca^2+^]_n_ modulation. Indeed, when we inhibited nuNCX1 with the chimeric fluorescent protein XIP-NLS, nuclear Ca^2+^ clearance decreased, whereas the [Ca^2+^]_n_ peak, elicited by ATP, increased. This effect on nucleoplasmic Ca^2+^ was due to the selective localization of XIP-NLS in the nucleus where it binded to the f-loop of nuNCX1, as also demonstrated by the coimmunoprecipitation between the peptide and nuNCX1 in isolated nuclei. Accordingly, the same interaction occurs at plasma membrane NCX level^22^. Interestingly, XIP-NLS inhibited nuNCX1 Ca^2+^_n_-extruding function without affecting the activity of plasma membrane NCX1, as demonstrated by patch-clamp experiments in whole neuronal cells. These data suggest that nuclear NCX1 plays a crucial role in regulating nuclear Ca^2+^ homeostasis upon [Ca^2+^]_i_ increase and works to remove nucleoplasmic Ca^2+^ accumulation in whole cell under physiological conditions.

Furthermore, the activity of nuNCX1 was involved in the regulation of PTEN/PI3K/Akt pathway, a transduction signal regulating neuronal differentiation. Moreover, during neuronal differentiation, the expression of PTEN, a phosphatase that converts phosphatidylinositol 3,4,5-trisphosphate to phosphatidylinositol 3,4-bisphosphate, was found to be inversely correlated to that of activated Akt. Specifically, the inhibition of nuNCX1 by XIP-NLS leaded to an increase in nuclear-resident PTEN expression and a decrease in Akt phosphorylation. Since nuNCX1, working in the forward mode, moves Ca^2+^ from nucleoplasm to NE lumen, it is conceivable that the blockade of this flux may promote, at nuclear level, a nucleoplasmic Ca^2+^-dependent activation of nuclear-resident PTEN. Indeed, a part the well-established role in antagonizing PI3’K signaling at the plasma membrane level, PTEN has been localized in the nucleus where it modulates transcriptional activity^32^. In addition, PTEN appears during elongation of growing neurites^33,34^, and increases progressively at the early stage of differentiation, possibly to prevent aberrant neurite extension. In our study, once upregulated, PTEN was able to reduce Akt phosphorylation, MAP-2 and GAP-43 expression, thus avoiding neuronal differentiation in cortical neurons. Another interesting finding of this study is that XIP-NLS increased the percentage of βIII-tubulin-positive immature neurons -after 7 DIV- in mature cultures of MAP-2-positive cortical neurons, thus suggesting that the selective inhibition of nuNCX1 hampered neuronal differentiation of cortical neurons. This is in line with a very recent paper showing that the chronic treatment with NCX inhibitors produce marked reduction of the Purkinje cell dendritic arbor^35^.

Collectively, this study unravels a new function for nuclear NCX1 within the complex regulatory machinery of nuclear calcium homeostasis, suggesting that this exchanger form may control neuronal differentiation through the fine regulation of Ca^2+^-dependent transduction mechanisms.

## Materials and methods

### Cell cultures

#### Clonal cells

PC12 cells were grown on plastic dishes in RPMI medium composed of 10% horse serum (HS), 5% fetal bovine serum (FBS), 100 UI/ml penicillin, and 100 μg/ml streptomycin. Neuronal differentiation was induced by exposing PC12 cells to NGF (50 ng/ml) were cultured in a humidified 5% CO_2_ atmosphere. Culture medium was changed every 2 days. For functional studies, cells were seeded on glass coverslips (Fisher, Springfield, NJ, USA), coated with poly-L-lysine (5 μg/ml) (Sigma, St. Louis, Missouri, USA), and used at least 12 h after seeding.

#### Embryonic neurons

cortical pure neurons were prepared from brains of 16-day-old Wistar rat embryos. Briefly, the rats were first anesthetized and then decapitated to minimize pain and distress. Dissection and dissociation were performed in Ca^2+^/Mg^2+^-free PBS containing glucose (30 mM). Tissues were incubated with papain for 10 min at 37 °C and dissociated by trituration in Earle’s Balanced Salt Solution containing DNase, bovine serum albumin (BSA), and ovomucoid. Cells were plated at 15 × 10^6^ in 100-mm plastic Petri dishes pre-coated with poly-D-lysine (20 μg/ml) in minimum Eagle’s medium/F12 (Life Technologies, Milan, Italy) containing glucose, 5% deactivated FBS, 5% deactivated HS (Life Technologies, Milan, Italy), glutamine, and antibiotics. Cytosine β-D-arabinofuranoside hydrochloride (Ara-C; 10 μM) was added within 48 h of plating to prevent non-neuronal cell growth. Neurons were cultured at 37 °C in a humidified 5% CO_2_ atmosphere and used after 7 days of culture. All experiments on primary cortical neurons were performed according to the procedures described in experimental protocols approved by the ethical committee of the “Federico II” University of Naples, Italy.

### Isolation of intact nuclei

Highly purified nuclei were obtained from cell cultures by the combined use of hypotonic shock and mechanical disruption. Briefly, when they reached confluence, cells were washed twice in saline phosphate buffer and lysed in hypotonic buffer (10 mM Tris-HCl, pH 7.8, 10 mM β-mercaptoethanol, 0.5 mM phenylmethylsulfonyl fluoride (PMSF), 1 μg/ml aprotinin, leupeptin, and pepstatin). After 20 minutes on ice, the swollen samples were mechanically disrupted. The nuclear pellet was obtained by centrifugation at 500 g for 6 min at 4 °C and was then washed in a solution containing 10 mM Tris-HCl, 2 mM MgCl2 plus protease inhibitors, pH 7.2. Finally, the nuclear pellet was resuspended in a standard solution (KCl 125 mM, HEPES 50 mM, K2HPO4 2 mM, EGTA 0.1 mM, CaCl2 0.3 mM, NaCl 3 mM, containing 1 mM ATP, pH 7.4)^11,34^. Nuclear preparations were used or stored at −80 °C until use. To check nuclear integrity, isolated nuclei were plated on glass coverslips (Fisher, Springfield, NJ, USA) previously coated with poly-L-lysine (30 µg/ml), and, then, fixed with 4% PAF (w/v) for 20 min at RT. After fixing, nuclear preparations were stained with ethidium bromide and/or Nissl solution to study the purity, integrity, and possible ER contamination of nuclear preparations.

### Inner and outer nuclear membranes separation

Freshly isolated nuclei of PC12 cells and cortical neurons were employed for isolation of NE as described previously^18^. Nuclear preparations were treated with 2% Na-citrate (trisodium, dihydrate) in a Nuclear Resuspension Buffer containing (in mM): 10 HEPES pH 7.9, 10 KCl, 1.5 MgCl_2_, 0.1 EGTA, 0.5 DTT, 1 sodium orthovanadate (Na_3_VO_4_), 0.2 PMSF, 350 sucrose, protease inhibitors. Nuclei were pelleted by brief centrifugation at 5000×g: the pellet containing inner nuclear membranes was resuspended in nuclear resuspension buffer, while the supernatant containing outer nuclear membranes was precipitated with 10% trichloroacetic acid^36^.

### Nuclear/cytosolic fractionation and western blot analysis

Cytosolic and nuclear fractions were obtained from NGF-differentiated PC12 cells. In brief, cells were washed twice and collected in saline phosphate buffer by centrifugation at 1500 rpm for 3 min. Then, the pellet was dissolved in a buffer containing (in mM) 10 Hepes pH 7.9, 10 KCl, 1.5 MgCl_2_, 0.1 EGTA, 0.5 DTT, 1 sodium orthovanadate (Na_3_VO_4_), 0.2 PMSF, and protease inhibitors; the cytosolic fraction was then obtained by two consecutive centrifugations at 3000 rpm for 5 min at 4 °C. Nuclear fractions were obtained dissolving the pellet in a solution containing (in mM) 20 Hepes pH 7.9, 400 NaCl, 1.5 MgCl_2_, 0.1 EGTA, 25% glycerol, 1 Na_3_VO_4_, 0.2 PMSF and protease inhibitors; throughout the procedure, the samples were kept on an agitation system for 30 min at 4 °C. Finally, samples were centrifuged at 13,000 rpm for 15 min.

For Western blot analysis, cell cultures, inner and outer nuclear membranes, and nuclear preparations were lysed with a buffer containing 20 mM tris–HCl (pH 7.5), 10 mM NaF, 1 mM phenylmethylsulfonyl fluoride, 1% NONIDET P-40, 1 mM Na_3_VO_4_, 0.1% aprotinin, 0.7 mg/ml pepstatin, and 1 μg/ml leupeptin. Samples were cleared by centrifugation and supernatants were used for Western blot analysis. Protein concentration in supernatants was determined by the Bradford method^37^. Protein samples (50 μg) were analyzed on 8% sodium dodecyl sulfate polyacrylamide gel with 5% sodium dodecyl sulfate stacking gel (SDS-PAGE) and electrotransferred onto Hybond ECL nitrocellulose papers (GE Healthcare, Little Chalfont, UK). Membranes were blocked with 5% non-fat dry milk in 0.1% Tween 20 (TBS-T; 2 mmol/l Tris–HCl, 50 mmol/l NaCl, pH 7.5) for 2 h at RT. Subsequently, they were incubated overnight at 4 °C in the blocked buffer containing the following antibodies: 1:1000 anti-NCX1 (rabbit polyclonal antibody, Swant, Bellinzona, Swiss), 1:1000 antibody for β-actin (mouse monoclonal antibody, Santacruz Biotechnology, Inc. CA), 1:500 anti-pAkt (mouse monoclonal antibody, Cell Signaling, Inc., MA, USA), 1:1000 anti-Akt (rabbit polyclonal antibody, Santa Cruz Biotechnology, Inc., CA, USA), 1:1000 anti-CRM1 (goat polyclonal antibody, Santa Cruz Biotechnology, Inc, CA, USA), 1:1000 anti-calretinin (rabbit polyclonal antibody; GeneTex, CA, USA), 1:500 anti-GAP-43 (mouse monoclonal antibody, Millipore Corporation, MA, USA), 1:1000 anti-MAP-2 (mouse monoclonal antibody, Sigma-Aldrich, Milan, Italy), 1:1000 anti-PTEN (mouse monoclonal antibody, Santa Cruz Biotechnology, Inc., CA, USA) and 1:2000 anti-α-tubulin (mouse monoclonal antibody, Sigma-Aldrich, Milan, Italy). Membranes were blocked with 5% non-fat dry milk in 0.1% Tween 20 (TBS-T; 2 mmol/l tris–HCl, 50 mmol/l NaCl, pH 7.5) for 2 h at RT. Membranes containing nuclear fractions from neuronal cells treated with XIP-NLS were incubated overnight at 4 °C in the blocked buffer containing the following antibodies: 1:1000 anti-SOD1 (rabbit polyclonal anti-body, Santa Cruz Biotechnology, Inc., CA, USA), 1:1000 anti-GFP (mouse monoclonal anti-body, Abcam, Cambridge, UK), 1:1000 anti-CREB (mouse monoclonal anti-body, Cell Signaling Technology, Inc. MA, USA) and pCREB (rabbit polyclonal anti-body, Millipore Corporation, MA, USA).

Membranes containing inner and outer nuclear fractions were incubated overnight at 4 °C in the blocked buffer containing the following antibodies: 1:1000 anti-NCX1 (rabbit polyclonal antibody, Swant, Bellinzona, Swiss); 1:200 anti-Lamin B1/B2 (mouse monoclonal antibody, Abcam, Cambridge, UK), 1:1000 calretinin (rabbit polyclonal antibody, GeneTex, Inc., GA, USA), and 1:1000 Karyopherin β2 (mouse monoclonal antibody, Santa Cruz Biotechnologies, Inc., CA, USA). Then, membranes were washed with 0.1% Tween 20 and incubated with the secondary antibodies (1:2000; GE Healthcare, Milan, Italy) for 1 h. Immunoreactive bands were detected using ECL reagent kits (GE Healthcare, Milan, Italy). The optical density of the bands was determined by Chemi-Doc Imaging System (Bio-Rad Laboratories, Milan, Italy).

### qRT-PCR analysis

 qRT-PCR was performed in a 7500-fast real-time PCR system (Applied Biosystems) by Fast SYBR Green Master Mix (Applied Biosystems). Samples were amplified simultaneously in triplicate as follows: heating 2 min at 50 °C, denaturation 10 min at 95 °C, amplification and quantification 35 cycles of 15 s at 95 °C; 1 min at 60 °C with a single fluorescence measurement. The data were normalized by using hypoxanthine phosphor-ribosyl-transferase (HPRT) as an internal control. Differences in mRNA content between groups were expressed by using 2−ΔΔCt.formula. The oligonucleotide sequences were for NCX1, forward: AGATTCCGTGACTGCAGTTGTG and reverse: ATACTGGTCCTGGGTAGCTGCTA; for NCX3, forward: CGGTCACAGCTGTTGTTTTTGT and reverse: CAGGGCAGCAGCTTTGCT; for HPRT, forward: TGGAAAGAACGTCTTGATTGTTGA and reverse: GCTGTACTGCTTGACCAAGGAA.

### Small interfering RNA

NCX1 knock down was achieved with siRNA duplexes against NCX1 and its non-targeting control (Invitrogen, San Giuliano Milanese, Milan, IT). The following gene-specific sequences were used successfully:

(RNA)-CCAAAUGGAGAGACCACCAAGACU and (RNA)-UAGUCUUGGUGGUCUCUCCAUUUGG or (RNA)-ACAUGUUCCUCGGAGUUUCUAUUAU and (RNA)-AUAAUAGAAACUCCGAGGAACAUGU. After 5 days in culture with NGF, PC12 cells were transfected for 5 h with each duplex at a final concentration of 10 nM using HyPerFect transfection reagent (Qiagen, Milan, IT).

### Generation and expression of XIP-NLS

The expressing vector encoding for XIP-NLS protein was obtained by sub-cloning in frame 2 copies of each XIP and NLS cDNAs in EYFP-C3 plasmid (Clontech Laboratories, Mountain View, CA), as shown in Fig. 4 panel A. In particular, 2 copies of the cDNA (from rat NCX1.1 NM_019268.3) encoding for XIP domain (DRRLLFYKYVYKRYRAGKQR) were obtained by PCR on a single plasmid and digested by specific restriction enzymes, whereas the two cDNAs encoding for NLS (from SV40 Large T-anti-gen) and a translational stop codon (PKKKRKV*) were obtained by in vitro annealing of two synthetic oligodeoxy nucleotide strains (Eurofinsgenomics, Ebersberg, Germany). Both XIP and NLS cDNAs were sequentially ligated in frame in the multicloning site of the EYFP-C3 plasmid. Two or more neutral aminoacids separated EYFP, XIP, and NLS domains. Successful construction of the plasmid encoding for YFP-XIP-NLS was verified by sequencing both strands (Microgem, Naples, Italy). This plasmid named XIP-NLS was transiently transfected in PC12 cells with Lipofectamine 2000 (Invitrogen, Carlsbad, CA), according to the manufacturer’s protocol. The efficiency of transfection was calculated by counting the number of EYFP-positive nuclei compared to total Hoecth-positive nuclei.

### Immunoprecipitation analyses

Cortical neurons were transfected with an excess of XIP-NLS (400 ng/ml). After transfection, nuclei from each population were isolated as previously described and homogenized in lysis buffer containing: 50 mM HEPES, 100 mM NaCl, 1.5 mM MgCl2, 1 mM PMSF, 0.2% Nonidet P-40, 5 μg/ml aprotinin, 10 μg/ml leupeptin and 2 μg/ml pepstatin. The nuclear lysates were cleared by centrifugation at 12,000 rpm for 10 min. At the end, 1 mg of nuclear lysate was immunoprecipitated with the mouse monoclonal anti-EGFP antibody (1:200) or the rabbit polyclonal anti-NCX1 antibody (1:200). Then, the immunoprecipitates were resolved by Western Blotting and immunoblot analysis was performed using anti-NCX1 or anti-GFP antibodies, respectively.

### Immunocytochemistry

Isoform-specific antibodies for NCX1 and Lamin B1/B2, alone or in combination, were used for immunocytochemistry. The rabbit polyclonal anti-NCX1 antibody was purchased from Swant (Bellinzona, Switzerland). Cells or isolated nuclei were rinsed twice in cold 0.01 M saline phosphate buffer (PBS) at pH 7.4 and fixed in 4% (w/v) paraformaldehyde (Sigma, Milan, Italy) for 20 min at RT. Following three washes in PBS, cells were blocked with 3% (w/v) BSA and 0.05% Triton-X (Biorad, Milan, Italy) for 1 h at RT. The coverslips were then incubated overnight with the primary antibody anti-NCX1 (1:1000 dilution), or anti-Lamin B1/B2 (1:50, Abcam, UK). After three washes in PBS, the coverslips were incubated in the dark for 1 h at RT with two secondary antibodies: Cy2 anti-mouse IgG and Cy3 anti-rabbit IgG (1:200, Jackson Immuno Research Laboratories, Inc. PA, USA). The immunosignal of NCX1 shown in Fig. 1 panel A and Fig. 2 panel A was detected by using a biotinylated secondary antibody. After this latter incubation, the peroxidase reaction was developed using 3,3′-diaminobenzidine/4-HCl as a chromogen. Then, after the final wash, coverslips were mounted with Vectashield (Vector Labs, Burlingame, CA). Fluorescence intensity of MAP2 and βIII-tubunin in cortical neurons was quantified in terms of pixel intensity value by using the NIH image software. Briefly, digital images were taken with 63x objective and identical laser power settings and exposure times were applied to all the photographs from each experimental set. The quantification of colocalization between MAP2 and βIII-tubun was assessed by using the ‘co-localization highlighter’ plug-in for ImageJ Software (NIH, Bethesda, MA, USA). Before colocalization analysis, threshold settings for each image were determined, and quantification was achieved by counting the number colocalized points per microscope field. Results were expressed as a percentage of colocalization. All images were observed using a Zeiss LSM510 META/laser-scanning confocal microscope.

### [Ca^2+^]_i_ and nuclear Ca^2+^ measurements

[Ca^2+^]_i_ was measured by single-cell computer-assisted video-imaging in isolated nuclei from NGF-differentiated PC12 loaded with Fura-2/AM (Calbiochem)^38,39^. Under these conditions, Fura-2 is trapped within the NE lumen. However, nucleoplasmic Ca^2+^ was measured in whole cells by loading NGF-differentiated PC12 with Fura-1dextran (10 μM/1 h at 37 °C in the presence of 0.05% Pluronic), a conjugated form of the fluorescent probe that is trapped in the nucleoplasmic space.

To perform functional analysis of Ca^2+^ level in the lumen of NE on isolated nuclei, we modified the methods used by Gerasimenko et al.^11^ and Xie et al.^18^. Fresh nuclear preparations from NGF-differentiated PC12 cells were placed on glass coverslips previously treated with 30 µg/ml poly-L-lysine. After 1 h, the preparation was loaded with 10 µM Fura-2/AM at 4 °C in a standard solution containing 0.04% Pluronic. At the end of the Fura-2/AM loading period, isolated nuclei were allowed to settle for 15 min in a TM solution composed of: 20 mM Tris-HCl, 1 mM MgCl2, 150 mM NaCl with a pH of 7.4 and then subjected to an uptake buffer named Na^+^-free consisting of: 250 mM sucrose, 50 mM Tris–HCl, 4 mM K2HPO4, 4 mM MgCl2, 2 mM EGTA, 2 mM EDTA, and 147 mM NMDG^+^ together with 2.5 mM CaCl2, pH 7.4^18^. To measure Ca^2+^ levels, both in whole cells and isolated nuclei, coverslips were placed into a perfusion chamber (Medical System, Co. Greenvale, NY, USA) mounted onto a Zeiss Axiovert 200 microscope (Carl Zeiss, Germany) equipped with a FLUAR 40X oil objective lens. The experiments were carried out with a digital imaging system composed of MicroMax 512BFT cooled CCD camera (Princeton Instruments, Trenton, NJ, USA), LAMBDA 10-2 filter wheel (Sutter Instruments, Novato, CA, USA), and Meta-Morph/MetaFluor Imaging System software (Universal Imaging, West Chester, PA, USA). After loading, nuclei were alternatively illuminated at wavelengths of 340 and 380 nm by a Xenon lamp. The emitted light was passed through a 512 nm barrier filter. Fura-2 fluorescence intensity was measured every 3 s and reported as F340/F380 ratios.

For the experiments of Figs. 6 and 7, NGF-differentiated PC12 cells and cortical neurons at 7 DIV were illuminated at wavelengths of 480 nm to identify EYFP-positive cells and then at 340 and 380 nm to measure, in the EYFP-positive cells, Fura-dextran-detected nucleoplasmic Ca^2+^ levels. Ca^2+^ levels have been reported as 340/380 ratio.

### Electrophysiological recording of NCX and voltage-gated sodium channel activity by patch clamp

The membrane voltage and current were acquired using pClamp10 software.

Plasma membrane NCX currents (I_NCX_) in NGF-differentiated PC12 cells were recorded by the patch-clamp technique in whole-cell configuration using the commercially available amplifier Axopatch200B and Digidata1322A interface (Molecular Devices), as previously described^28,40^. I_NCX_ were recorded starting from a holding potential of −60 mV up to a short-step depolarization at +60 mV (60 ms). A descending voltage ramp from +60 mV to −120 mV was applied. I_NCX_ recorded in the descending portion of the ramp (from +60 mV to −120 mV) were used to plot the current–voltage (I–V) relation curve. The I_NCX_ magnitude was measured at the end of +60 mV (reverse mode) and at the end of −120 mV (forward mode), respectively. The Ni^2+^-insensitive component was subtracted from total currents to isolate I_NCX_^21,41^. Neuronal cells were perfused with external Ringer’s solution containing the following (in mM): 126 NaCl, 1.2 NaHPO4, 2.4 KCl, 2.4 CaCl2, 1.2 MgCl2, 10 glucose, and 18 NaHCO3, pH 7.4. Twenty millimolar tetraethylammonium (TEA), 50 nM TTX, and 10 μM nimodipine were added to Ringer’s solution to abolish potassium, sodium, and calcium currents. The dialyzing pipette solution contained the following (in mM): 100 K-gluconate, 10 TEA, 20 NaCl, 1 Mg-ATP, 0.1 CaCl2, 2 MgCl2, 0.75 EGTA, and 10 HEPES, adjusted to pH 7.2 with CsOH.

For tetrodotoxin (TTX)-sensitive Na^+^ channel recordings, NGF –differentiated PC12 cells and untreated PC12 cells were perfused with an extracellular Ringer’s solution containing 20 mM tetraethylammonium (TEA) and 5 μM nimodipine. The pipettes were filled with 110 mM CsCl, 10 mM TEA, 2 mM MgCl2, 10 mM EGTA, 8 mM glucose, 2 mM Mg-ATP, 0.25 mM cAMP, and 10 mM HEPES (pH 7.3). TTX-sensitive Na^+^ currents were recorded as previously described by Secondo et al^23^. The mean resting membrane potential (holding potential at 0 current) was measured using the above-mentioned bath solution in absence of TTX and TEA. Capacitive currents were estimated from the decay of the capacitive transient induced by 5 mV depolarizing pulses from a holding potential of −80 mV and acquired at a sampling rate of 50 kHz. The capacitance of the membrane was calculated according to the following equation: Cm- = τc •Io/ΔEm (1−I∞/Io), where Cm is membrane capacitance, τc is the time constant of the membrane capacitance, Io is the maximum capacitance current value, ΔEm is the amplitude of the voltage step, and I∞ is the amplitude of the steady state current.

### Statistical analysis

Data are expressed as mean ± S.E.M. Statistical comparisons between controls and treated experimental groups were performed using the one-way ANOVA, followed by Newman Keul’s test. *p* < 0.05 was considered statistically significant.
