# Author Correction: Na^+^/Ca^2+^ exchanger 1 on nuclear envelope controls PTEN/Akt pathway via nucleoplasmic Ca^2+^ regulation during neuronal differentiation

**DOI:** 10.1038/s41420-019-0162-x

**Published:** 2019-03-27

**Authors:** Agnese Secondo, Alba Esposito, Tiziana Petrozziello, Francesca Boscia, Pasquale Molinaro, Valentina Tedeschi, Anna Pannaccione, Roselia Ciccone, Natascia Guida, Gianfranco Di Renzo, Lucio Annunziato

**Affiliations:** 1 Division of Pharmacology, Department of Neuroscience, Reproductive and Odontostomatological Sciences, School of Medicine, “Federico II” University of Naples, Naples, Italy; 2 IRCCS SDN, Naples, Italy

**Correction to:** **Cell Death Discovery**

10.1038/s41420-017-0018-1

published online 30 April 2018

Following publication of the original article^1^, it was brought to the attention of the Editors that there had been an error in the author affiliations We apologize for any inconvenience this may have caused readers. The correct affiliations are as follows:

Agnese Secondo^1^, Alba Esposito^1^, Tiziana Petrozziello^1^, Francesca Boscia^1^, Pasquale Molinaro^1^, Valentina Tedeschi^1^, Anna Pannaccione^1^, Roselia Ciccone^1^, Natascia Guida^2^, Gianfranco Di Renzo^1^ & Lucio Annunziato^2^

1 Division of Pharmacology, Department of Neuroscience, Reproductive and Odontostomatological Sciences, School of Medicine, “Federico II” University of Naples, Italy

2 IRCCS SDN, Naples, Italy
